# Precise genome editing without exogenous donor DNA via retron editing system in human cells

**DOI:** 10.1007/s13238-021-00862-7

**Published:** 2021-08-17

**Authors:** Xiangfeng Kong, Zikang Wang, Renxia Zhang, Xing Wang, Yingsi Zhou, Linyu Shi, Hui Yang

**Affiliations:** 1 HUIGENE Therapeutics Inc., Shanghai, 200131 China; 2 Institute of Neuroscience, State Key Laboratory of Neuroscience, Key Laboratory of Primate Neurobiology, Center for Excellence in Brain Science and Intelligence Technology, Chinese Academy of Sciences, Shanghai, 200031 China; 3 College of Life Sciences, University of Chinese Academy of Sciences, Beijing, 100049 China; 4 Shanghai Center for Brain Science and Brain-Inspired Intelligence Technology, Shanghai, 201210 China

**Dear Editor**,

CRISPR-Cas9 mediated seamless genome editing can be achieved by incorporating donor DNA into the CRISPR-Cas9 target loci via homology-directed repair (HDR), albeit with relative low efficiency due to the inefficient delivery of exogenous DNA (Cox et al., 2015; Gao, 2021). Retrons are bacterial phage-defense related operons composed of a specialized reverse transcriptase (RT) and a relevant non-coding RNA (ncRNA) which can be partially reverse transcribed by RT initiating at a conserved guanosine (G) residue to produce a multicopy single-stranded DNA (msDNA) (Yee et al., 1984; Millman et al., 2020). After being reverse transcribed, the msDNA is usually covalently tethered to the ncRNA through the 2′,5′-phosphodiester bond between the priming G in ncRNA and 5′ end of msDNA (Dhundale et al., 1987). The reverse transcription process, of which the specialized RT recognizes the unique secondary structure of retron ncRNA, is highly specific (Hsu et al., 1989). Additionally, desired msDNA can be generated *in vivo* by replacing the dispensable region of retron ncRNA with desired sequences (Mirochnitchenko et al., 1994; Simon et al., 2019). Therefore, retrons are promising biological sources for *in vivo* generation of DNA donors for HDR-mediated precise genome editing.

Recently, several studies have shown that retrons coupled with CRISPR-Cas9 could enhance precise genome editing via HDR in bacterium and yeast through fusing guide RNA (gRNA) to the 3′ end of retron ncRNA, producing multicopy single-stranded DNA (msDNA) covalently tethered to gRNA (Sharon et al., 2018; Lim et al., 2020; Schubert et al., 2021). In this study, we further engineered retrons by fusing Cas9 with *E. coli* RT from different clades and joining gRNA at the 5′ end of retron ncRNA, and found that retron editing can achieve precise genome editing efficiently in human cells. By co-expression of Cas9-RT fusions and retron-ncRNA gRNA (rgRNA) in HEK293T cells, we demonstrated the rate of retron editing-mediated HDR events at endogenous genomic loci was up to 10%. We expect our retron editing system could aid in advancing the *ex vivo* and *in vivo* therapeutic applications of retrons.

Given that HDR-mediated precise editing can be enhanced by increasing the local abundance of donor DNA, and CRISPEY strategy showed highly efficient editing in yeast genome by *in situ* expressing retron-gRNA chimeric molecule (Sharon et al., 2018). Therefore, we attempted to *in situ* reverse transcribe the donor msDNA covalently tethered to gRNA by fusing retron ncRNA at the 5′ or 3′ end of gRNA, termed as the retron-ncRNA gRNA (rgRNA) (Fig. 1A and 1B). Four experimentally validated *E. coli* retrons were evaluated for *in vivo* production of msDNA in human cells (Figs. 1B and S1). Additionally, we fused retron RT to the amino terminus or carboxy terminus of Cas9 with XTEN linker to increase the spatial proximity between RT and Cas9, which may enhance the retron editing in human cells by increasing the abundance of donor msDNA in the vicinity of DSB stimulated by Cas9 (Fig. 1B).

**Figure 1:**
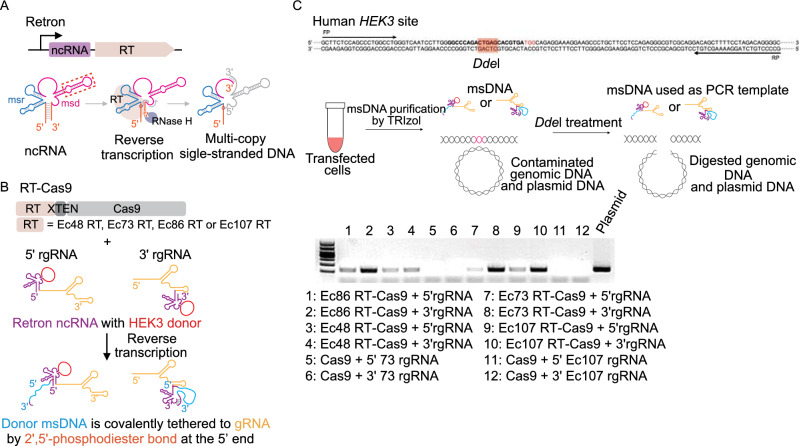
**Retron-mediated generation of msDNA in human cells.** (A) Schematic representing retron-mediated msDNA production. Retrons are composed of non-coding RNA (ncRNA) and a specific reverse transcriptase (RT). The msr and msd region of retron ncRNA form secondary structure which can be specifically recognized by relevant retron RT reverse transcribing part of msd region, generating multicopy single-stranded DNA (msDNA). After reverse trancription, the msd template is degraded by RNase H. The replaceable region of ncRNA, in which the donor sequence is inserted, is marked by red-dotted box. Red circle indicates 2′,5′-phosphodiester bond between 2′ end of priming guanosine (G) and 5′ end of the msDNA. (B) Plasmids construction strategies for retron-mediated generation of msDNA in human cells. Human codon-optimized RT-XTEN-spCas9 fusions are driven by the CAG promoter, whereas the 5′ extended retron-ncRNA gRNA (5′ rgRNA), in which the retron ncRNA is fused to the 5′ of gRNA, or 3′ rgRNA, is driven by the EF1α promoter. Four *E*. *coli* retrons (Ec48 RT, Ec73 RT, Ec86 RT and Ec107 RT) from different clades were used in our retron editing system. The replaceable region of retron ncRNA was replaced by a 122 nt modified HEK3 sequencce. (C) Determination of msDNA level. The msDNA abundance was determined by PCR. PCR was conducted using the *DdeI* digested product as template

We first sought to test the relative abundance of msDNA in human cells. We found all of four selected RTs enabled the expression of msDNA in human cells, and retron RT combining with 3′ rgRNA showed higher expression of msDNA comparing to that with 5′ rgRNA (Fig. 1C). The results indicated that retron RT were reverse transcription-functional in human cells, inspiring us to further study the potential of retron editing at human endogenous genomic loci.

Next, we investigated the potential of retron editing in human HEK293T cells (Figs. 2 and S2). We transfected the HEK293T cells with plasmids expressing different Cas9-RT fusions targeting *EMX1* locus and *HEK3* site (Anzalone et al., 2019), and plasmids expressing 5′ rgRNA or 3′ rgRNA that can produce msDNA with 120 nucleotide (nt)-long homology donor sequences as well (Fig. 2A and 2B). To determine the retron editing-mediated HDR efficiency at *EMX1* and *HEK3*, we performed deep sequencing. Among four retrons tested, Ec86, Ec73 and Ec107 achieved varying degrees of precise editing and Ec73 combining with CRISPR-Cas9 showed highest activity, up to 10% (Fig. 2C). Restriction-fragment length polymorphism (RFLP), confirmed the deep sequencing results (Fig. S2A). In addition, retron editing with shortened homology donor sequences (90 nt) also enabled precise editing, including insertions and transversions (Fig. S2B). To test that retron editing is RT activity-dependent, point mutations of D189A and D190A were introduced to the predicted active site of the Ec73 (Figs. 2D and S3), generating a catalytically dead Ec73 (dEc73). As expected, compared with Cas9 fused with wild-type Ec73, Cas9 fused with dEc73 or Cas9 only dramatically reduced the retron editing-mediated HDR efficiency (Fig. 2D). Together, our results indicated that Cas9-Ec73 RT fusion combining with 3′ Ec73 rgRNA can be harnessed for efficient precise genome editing in human cells (Fig. 2E). Of note, besides HDR events, we detected relatively high frequency of indels simulated by retron editing system (Figs. S2 and S4), suggesting Cas9-RT fusions co-expressing with relevant rgRNA retained the double stranded DNA cleavage activity of Cas9.

**Figure 2:**
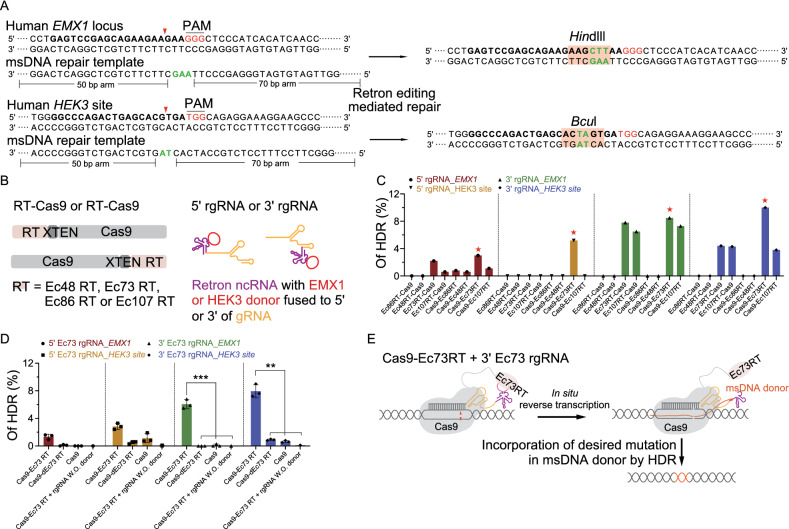
**Retron editing system mediates precise editing at endogenous genomic loci.** (A) Schematic of retron editing at human *EMX1* locus and *HEK3* site. The reverse-transcribed msDNA repair template complementary to the non-target strand is used as a repair template. The target sequence is in bold. The insertion sequence is marked in green letter. After retron editing, *Hin*dIII and *Bcu*I site is inserted into the *EMX1* locus and *HEK3* locus, respectively. (B) Plasmids construction strategies for retron editing system in human cells. Different Cas9-RT fusions targeting *EMX1* or *HEK3* combining with 5′ rgRNA or 3′ rgRNA that can produce msDNA with homology donor sequences relative to *EMX1* or *HEK3*, respectively, were co-expressed in HEK293T cells. (C) Analysis of retron editing efficiency by deep sequencing. (D) Deep sequencing to determine the efficiency of retron editing. dEc73 RT represents predicted catalytically dead Ec73 RT containing point mutation of D189A and D190A. rgRNA W.O. donor represents rgRNA without donor sequences inserted. ***P < 0.001, **P < 0.01, determined by Student’s *t*-test, *n* = 3. (E) Schematic diagram showing that Cas9-Ec73 RT fusion *in-situ* reverse transcribe 3′ Ec73 rgRNA to generate the msDNA containing a single-stranded donor sequence with homology arms, and msDNA is incorporated into the genome site cut by Cas9.

In addition, four of reported CRISPR-Cas9 off-target sites relative to *EMX1* locus were checked to determine the off-targeting effects of retron editing (Fig. S5). Although, Cas9-Ec73RT with rgRNA induced similar rate of indels at on-target sites as Cas9 with sgRNA (Fig. S5B), Cas9-Ec73RT with rgRNA showed reduced activity at the four known off-target sites, with 4.9-fold lower average frequency than that of Cas9 with sgRNA. To be noted, the off-targeting effects of msDNA should be checked before further application of retron editing, although the frequency of random insertion of ssDNA is much lower than that of dsDNA.

The ability to write any modification of interest into the genome is a long-sought goal of biotechnology. Retrons, capable of producing intracellular ssDNA as donor with high specificity, are promising biological sources for precise genome editing. In this study, we demonstrate that different retrons are functional in human cells. Moreover, by co-expressing Cas9-RT fusions and 3′ extended retron-ncRNA gRNA, retron editing could mediate precise genome editing in human cell. Compared with ssDNA-mediated HDR in 293T cells, no exogenous ssDNA donor is required in retron editing system.

Additionally, retrons coupled with CRISPR can efficiently insert a GFP gene in yeast with efficiency up to 87% (Sharon et al., 2018), making us anticipating retron editing a more versatile genome editor in both therapeutic applications and crop improvement. Precise genome editing on locus corresponding to agronomic traits can greatly accelerate the crop breeding (Mao et al., 2019; Gao, 2021). Although, base transversions, small insertion and deletion by base editors and prime editors have been successfully achieved in plant cells (Gao, 2021). However, HDR-mediated large fragment insertions cannot be efficiently achieved due to the low HDR efficiency and limit of exogenous donor DNA delivery in plant cells (Gao, 2021). Therefore, it is interesting to test the possibility of using retron editing for large fragment insertion in plant cells.

Many approaches may be done to improve retron editing in human cells. Firstly, identification of more suitable retron for genome editing in human cells. Second, engineered evolution of both retron-RT and retron-ncRNA can be done to enhance retron mediated intracellular production of ssDNA. Finally, evidences were shown that hRAD51 mutant fused to Cas9 (D10A) nickase (RDN) fusions could mediate precise genome editing without DSBs (Rees et al., 2019). It is plausible to anticipate that combination of retrons with RDN may make retron editing more accurate and safer due to the elimination of Cas9-induced DSBs.

To be noted, when we are preparing this manuscript, Zhao and colleagues preprinted their work of retron-mediated precise gene editing in human cells, which further convinced that retrons can be harnessed for precise genome editing in human cells by coupling with CRISPR-Cas9 (Zhao et al., 2021).

## Supplementary Information

Below is the link to the electronic supplementary material.

Supplementary file1 (PDF 3424 KB)
