# Supplementary material for: Precise genome editing without exogenous donor DNA via retron editing system in human cells
Source: Protein Cell. 2021 Aug 17;12(11):899–902. doi: 10.1007/s13238-021-00862-7 (PMC8563936; doi:10.1007/s13238-021-00862-7)
Supplement: Supplementary file 1 — Supplementary file1 (PDF 3424 KB) [file 13238_2021_862_MOESM1_ESM.pdf]

Supplemental materials

Materials and Methods

Plasmids construction

All DNA sequences of plasmids used in this study were provided in Supplemental Sequences. Human codon-optimized retron RTs were synthesized and inserted into EcoRI-digested pHG023 backbone or SmaI-digested pHG077 backbone by NEBuilder (New England Biolabs). The cloning of plasmids for expressing 5' rgRNA and 3' rgRNA were based on pHG215 backbone. Donor sequences were cloned and inserted into the replaceable region of retron ncRNA with *EcoRI* digestion and Gibson assembly using NEBuilder. Primers for cloning were listed in Supplemental Table1.

Cell Culture, transfection and flow cytometry sorting

Cell Culture, transfection and flow cytometry were performed as previously described (Xu et al., 2021). Briefly, HEK293T cells cultured in DMEM supplemented with 10% FBS and penicillin/streptomycin were seeded on 24-well poly-D-lysine coated plates (Corning). Transfection was conducted with 4 µl of PEI (Polyscience) following the manufacturer's manual and 1 µg of CR plasmids and 1 µg of rgRNA plasmids. Unless otherwise stated, transfected cells were sorted by MoFlo XDP 72 h after transfection. BFP and mCherry double-positive cells were collected.

msDNA purification and quantification

Four thousand sorted cells were centrifuged down at 7,000 rpm for 5 mins. Cells plate were resuspended in Trizol for isolating rgRNA-msDNA hybrid as the the manufacturer's manual for RNA purification. For quantifying the abundance of *HEK3* msDNA, the rgRNA-msDNA extract was digested by *DdeI* to eliminate the contamination of plasmid DNA and genomic DNA. PCR was conducted using the *DdeI* digested product as template.

Genomic DNA extraction

Four thousand sorted cells were harvested for genomic DNA extraction by addition of 10 µl of lysis buffer (Vazyme) following the manufacturer's manual.

Efficiency analysis of retron editing at human endogenous genomic loci

The genomic DNA extraction was performed as described above. FOR deep sequencing analysis, the genomic region in the vicinity of Cas9 target site was amplified by Phanta Max Super-Fidelity DNA Polymerase (Vazyme) using nested PCR: First round: 98 ℃, 3 mins; (98 ℃, 30 sec; 58 ℃, 30 sec; 72 ℃, 1 min) X 20 cycles; 72 ℃, 5 min. Second round: 98 ℃, 3 mins; (98 ℃, 30 sec;

56 ℃, 30 sec; 72 ℃, 30 sec) X 35 cycles; 72 ℃, 5 min; primers with barcode were used. PCR products were purified by Gel extraction kit (Vazyme) and sequenced on an Illumina HiSeq X System (150-bp paired-end reads). Forward reads were aligned to the reference sequences using BWA (v0.7.17-r1188) with parameter of "bwa mem -A2 -O3 -E1". At each target, editing was calculated as the percentage of total reads containing desired edits without indels within a 10-bp window of the cut site.

Restriction-fragment length polymorphism (RFLP) was performed to determine the CR-mediated precise editing. Purified PCR products were digested with *HindIII* and *BcuI* (Thermo) for *EMX1* and *HEK3* amplicon respectively, and incubated for 90 mins at 37 ℃. The digested products were analyzed by agarose gel electrophoresis and imaged with gel imaging system (Tanon).

All primers used for genomic DNA amplification are listed in Supplemental Table1.

Activity determination of retron editing at known Cas9 off-target sites

Determination was performed as previous (Anzalone et al., 2019). In brief, the genomic region of interest was amplified using nested PCR: First round: 98 ℃, 3 mins; (98 ℃, 30 sec; 58 ℃, 30 sec; 72 ℃, 1 min) X 20 cycles; 72 ℃, 5 min. Second round: 98 ℃, 3 mins; (98 ℃, 30 sec; 56 ℃, 30 sec; 72 ℃, 30 sec) X 35 cycles; 72 ℃, 5 min. Purified PCR products were Sanger sequenced and analyzed as previously described (Brinkman et al., 2018).

Supplemental References

Anzalone, A.V., Randolph, P.B., Davis, J.R., Sousa, A.A., Koblan, L.W., Levy, J.M., Chen, P.J., Wilson, C., Newby, G.A., Raguram, A., *et al.* (2019). Search-and-replace genome editing without double-strand breaks or donor DNA. Nature *576*, 149-+.

Brinkman, E.K., Kousholt, A.N., Harmsen, T., Leemans, C., Chen, T., Jonkers, J., and van Steensel, B. (2018). Easy quantification of template-directed CRISPR/Cas9 editing. Nucleic Acids Res *46*, e58.

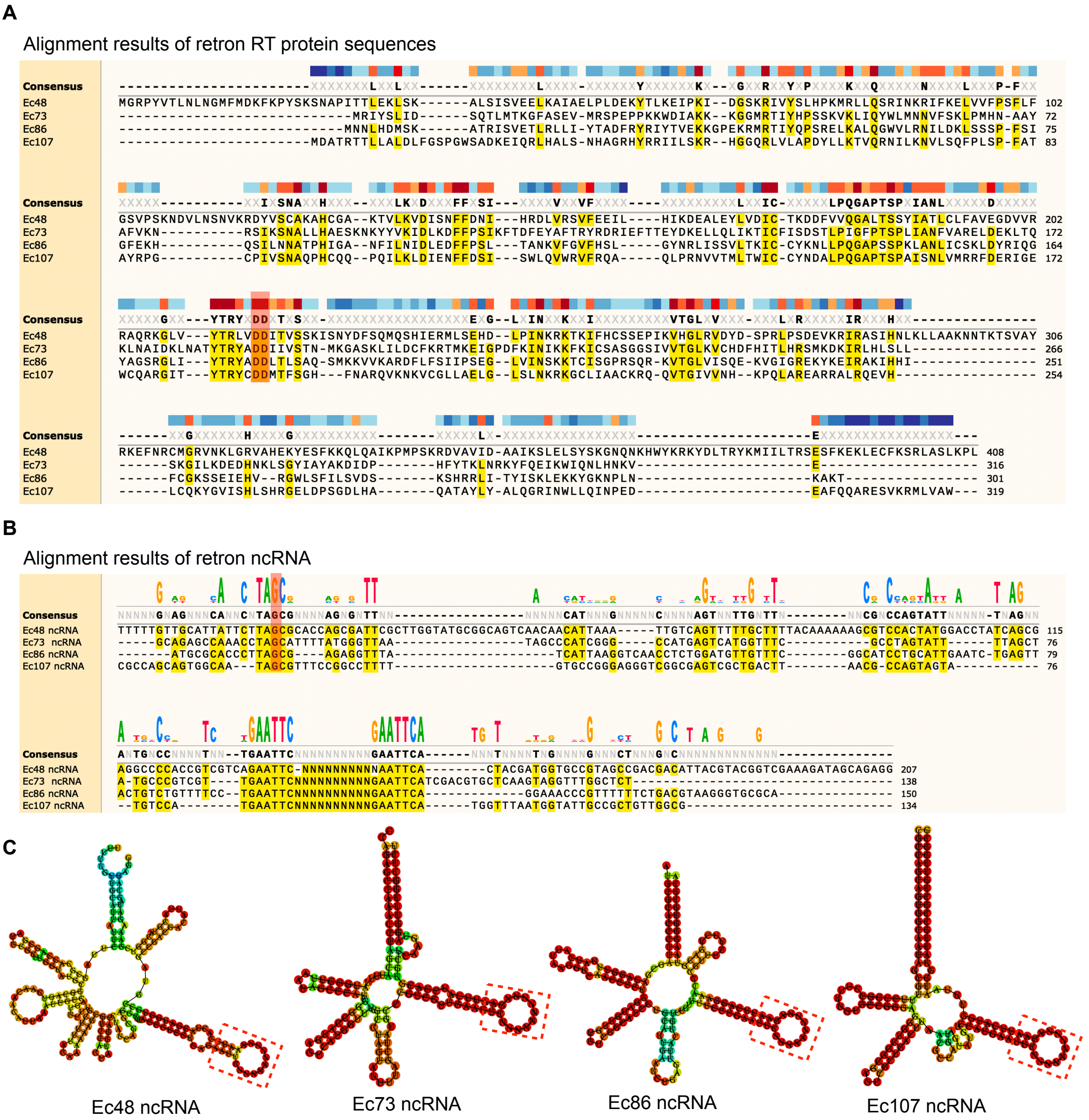

Supplemental Figure 1. Comparison of four retrons.
(A) Protein sequences alignment of Ec48-RT, Ec73-RT, Ec86-RT and Ec107-RT. Conserved double aspartic acid (DD) were highlighted in red. **(B)** Alignment of Ec48-ncRNA, Ec73-ncRNA, Ec86-ncRNA and Ec107-ncRNA. Conserved priming G was highlighted in red. **(C)** Predicted secondary structure of Ec48-ncRNA, Ec73-ncRNA, Ec86-ncRNA and Ec107-ncRNA using RNAfold web server. The replaceable region of retron ncRNA was marked with red dotted line.

A

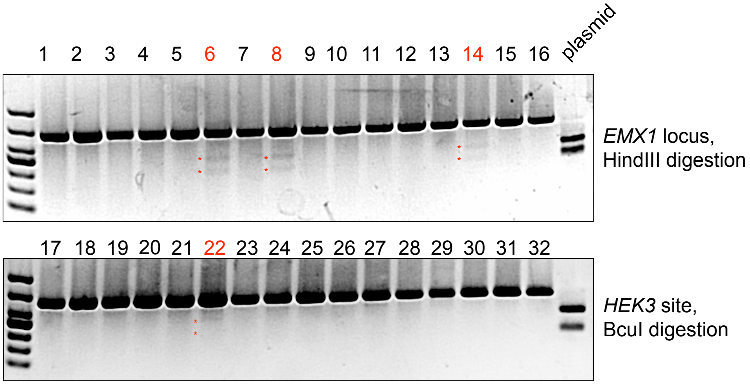

1/25: Ec86 RT-Cas9 + 5' Ec86 rgRNA
2/26: Ec86 RT-Cas9 + 3' Ec86 rgRNA
3/27: Ec48 RT-Cas9 + 5' Ec48 rgRNA
4/28: Ec48 RT-Cas9 + 3' Ec48 rgRNA
5/29: Ec73 RT-Cas9 + 5' Ec73 rgRNA
6/30: Ec73 RT-Cas9 + 3' Ec73 rgRNA
7/31: Ec107 RT-Cas9 + 5' Ec107 rgRNA
8/32: Ec107 RT-Cas9 + 3' Ec107 rgRNA

9/17: Cas9-Ec86 RT + 5' Ec86 rgRNA
10/18: Cas9-Ec86 RT + 3' Ec86 rgRNA
11/19: Cas9-Ec48 RT + 5' Ec48 rgRNA
12/20: Cas9-Ec48 RT + 3' Ec48 rgRNA
13/21: Cas9-Ec73 RT + 5' Ec73 rgRNA
14/22: Cas9-Ec73 RT + 3' Ec73 rgRNA
15/23: Cas9-Ec107 RT + 5' Ec107 rgRNA
16/24: Cas9-Ec107 RT + 3' Ec107 rgRNA

B

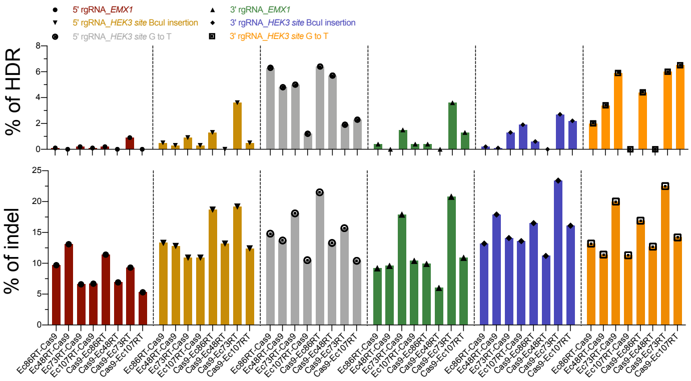

Supplemental Figure 2. Retron editing system-stimulated precise editing and indels. (A) Determination of retron editing system-mediated precise genome editing by restriction-fragment length polymorphism (RFLP) analysis. Digested products were marked by asterisks. **(B)** Efficiency determination of retron editing with 90nt donor by TIDER analysis. Transfected cells were directly collected without FACS sorting for genomic DNA extraction and TIDER analysis.

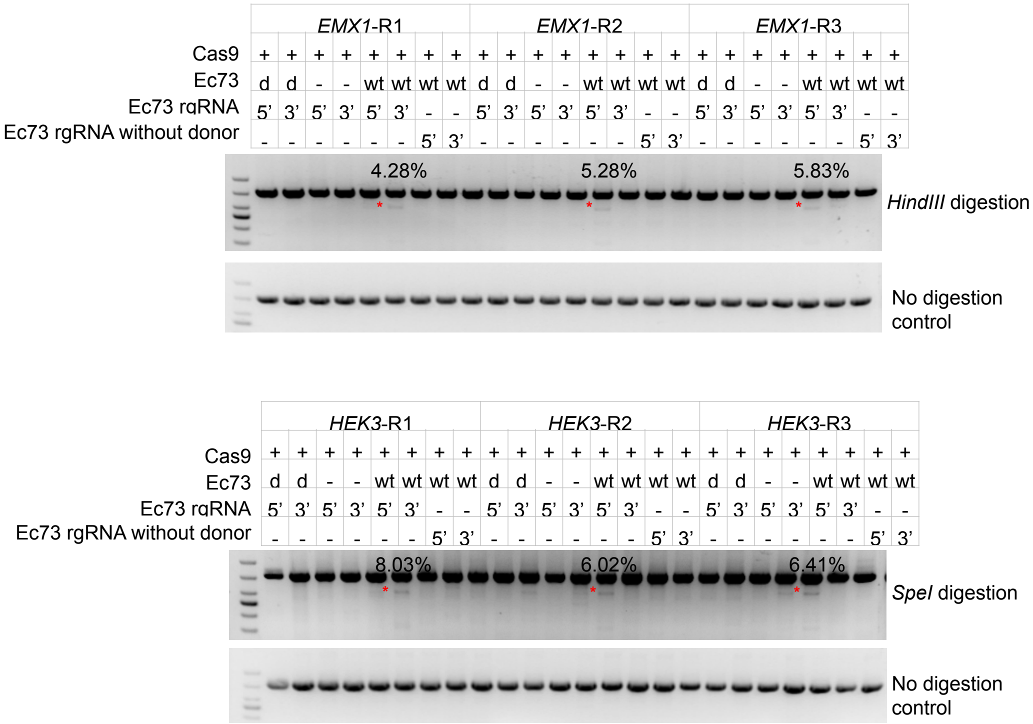

Supplemental Figure 3. RFLP analysis of retron editing system mediated precise editing.
Digested products were marked by asterisks. d represents predicted catalytically dead Ec73 RT, wt indicates wild-type Ec73 RT.

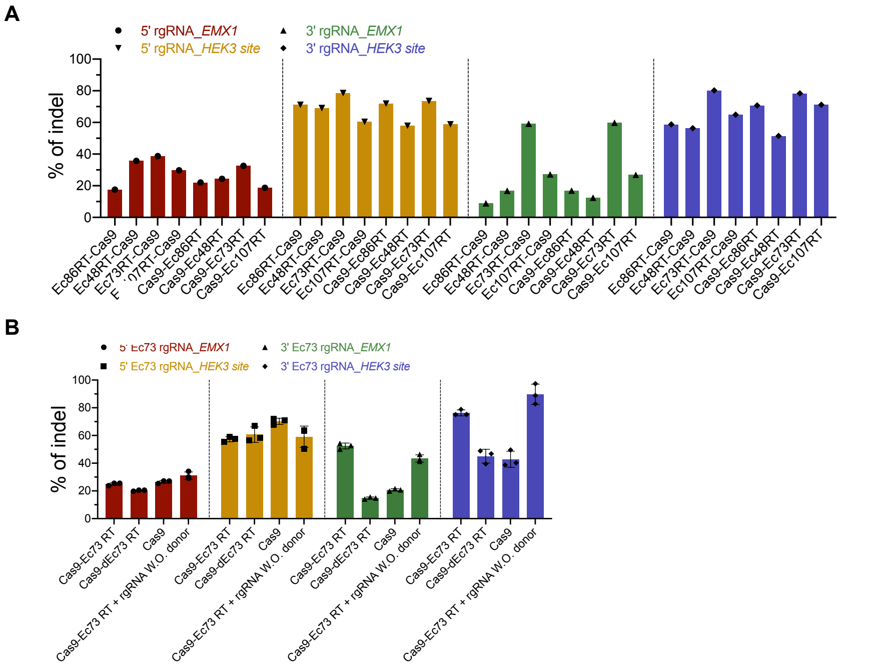

Supplemental Figure 4. Deep sequencing analysis of retron editing system induced indels.
(A) Related to Fig. 2C and **(B)** related to Fig. 2D

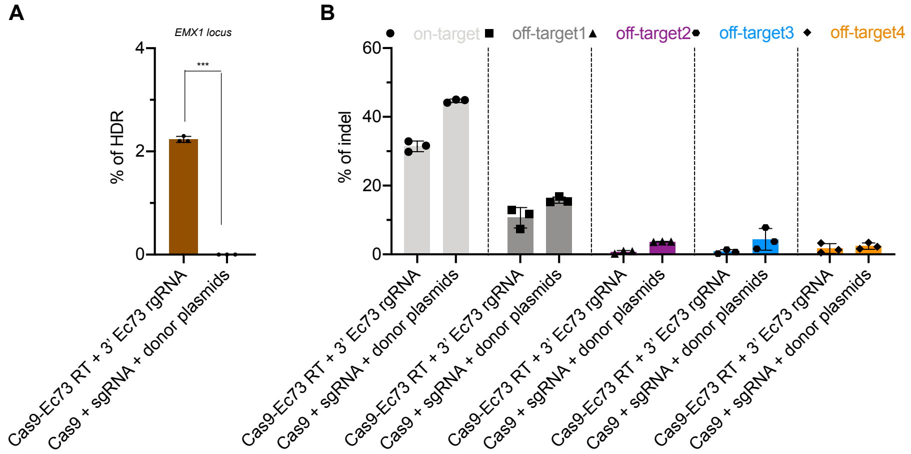

Supplemental Figure 5. Off target of retron editing at known Cas9 off target sites of *EMX1* locus.
(A) Retron editing-mediated HDR at *EMX1* locus. ***ρ < 0.001, determined by Student's t-test, n=3. **(B)** Retron editing-mediated indel at on-target site and four known Cas9 off target sites of *EMX1* locus, n=3. Transfected cells were directly collected after 3 days of transfection for genomic DNA extraction and TIDER analysis, with no FACS sorting.

Supplemental Sequences

Cas9-RTs fusion plasmids:

pCAGNLS-Cas9-NLS-XTEN-Ec86 RT-polyA-pCMV-BFP-polyA

[illegible]

pCAG-NLS-Cas9-NLS-XTEN-Ec48 RT-polyA-pCMV-BFP-polyA

[illegible]

pCAG-NLS-Cas9-NLS-XTEN-Ec73 RT-polyA-pCMV-BFP-polyA

[illegible]

Cas9-RTs fusion plasmids:

pCAG-NLS-Cas9-XTEN-Ec107 RT-NLS-polyA-pCMV-BFP-polyA

[illegible]

pCAG-NLS-Ec86 RT-XTEN-Cas9-NLS-polyA-pCMV-BFP-polyA

[illegible]

pCAG-NLS-Ec48 RT-XTEN-Cas9-NLS-polyA-pCMV-BFP-polyA

[illegible]

Cas9-RTs fusion plasmids:

pCAG-NLS-Ec73 RT-XTEN-Cas9-NLS-polyA-pCMV-BFP-polyA

[illegible]

pCAG-NLS-Ec107 RT-XTEN-Cas9-NLS-polyA-pCMV-BFP-polyA

[illegible]

rgRNA expression plasmids:

pEF1a-HH-Ec86 ncRNA-gRNA-HDV-polyA-pCMV-mCherry-polyA

[illegible]

pEF1a-gRNA-Ec86 ncRNA-HDV-polyA-pCMV-mCherry-polyA

[illegible]

pEF1a-HH-Ec48 ncRNA-gRNA-HDV-polyA-pCMV-mCherry-polyA

[illegible]

pEF1a-gRNA-Ec48 ncRNA-HDV-polyA-pCMV-mCherry-polyA

[illegible]

pEF1a-HH-Ec73 ncRNA-gRNA-HDV-polyA-pCMV-mCherry-polyA

[illegible]

pEF1a-gRNA-Ec73 ncRNA-HDV-polyA-pCMV-mCherry-polyA

[illegible]

rgRNA expression plasmids:

pEF1a-HH-Ec73 ncRNA-gRNA-HDV-polyA-pCMV-mCherry-polyA

ggctccggtgcccgtcagtgggcagagcgcacatcgcccacagtccccgagaagttggggggaggggtcggcaattgaaccggtgcctagagaaggtggcgcggggtaaactgggaaagtgatgtcgtgtactggctccgcctttttcccgagggtgggggagaaccgtatataagtgcagtagtcgccgtgaacgttctttttcgcaacgggtttgccgccagaacacaggtaagtgccgtgtgtggttcccgcgggcctggcctctttacgggttatggcccttgcgtgccttgaattacttccacctggctgcagta
cgtgattcttgatcccgagcttcgggttggaagtgggtgggagagttcgaggccttgcgcttaaggagccccttcgcctcgtgcttgagttgaggcctggcctgggcgctggggccgccgcgtgcgaatctggtggcaccttcgcgcctgtctcgctgctttcgataagtctctagccatttaaaatttttgatgacctgctgcgacgctttttttctggcaagatagtcttgtaaatgcgggccaagatctgcacactggtatttcggtttttggggccgcgggcggcgacggggcccgtgcgtcccagcgcacatgttcggcga
ggcggggcctgcgagcgcggccaccgagaatcggacgggggtagtctcaagctggccggcctgctctggtgcctggcctcgcgccgccgtgtatcgccccgccctgggcggcaaggctggcccggtcggcaccagttgcgtgagcggaaagatggccgcttcccggccctgctgcagggagctcaaaatggaggacgcggcgctcgggagagcgggcgggtgagtcacccacacaaaggaaaagggcctttccgtcctcagccgtcgcttcatgtgactccacggagtaccgggcgccgtccaggca
cctcgattagttctcgagcttttggagtacgtcgtctttaggttggggggaggggttttatgcgatggagtttccccacactgagtgggtggagactgaagttaggccagcttggcacttgatgtaattctccttggaatttgccctttttgagtttggatcttggttcattctcaagcctcagacagtggttcaaagtttttttcttccatttcaggtgtcgtgaacgcgtACTGCTGGCGctgatgagtccgtgaggacgaaacgagtaagctcgtcCGCCAGCAGTGGCAATAGCGTTTCCGGCCTTTTGTGC
CGGGAGGGTCGGCGAGTCGCTGACTTAACGCCAGTAGTATGTCCATgaattcATACCCAAAGgaattcATGGTTTAATGGTATTGCCGCTGTTGGCGAAATTAACAAACTAAggGTCTTCgaGAAGACCtgttttagagctagaaatagcaagttaaaataaggctagtccgttatcaacttgaaaaagtggcaccgagtcggtgcttttttttggccggcatggtcccagcctcctcgctggcgccggctgggcaacaTGcttcggCAtggcgaatgggaca
cgcgtCAGCCTCGACTGTGCCTTCTAGTTGCCAGCCATCTGTTGTTTGCCCCTCCCCCGTGCCTTCCTTGACCCTGGAAGGTGCCACTCCCACTGTCCTTTCCTAATAAAATGAGGAAATTGCATCGCATTGTCTGAGTAGGTGTCATTCTATTCTGGGGGGTGGGGTGGGGCAGGACAGCAAGGGGGAGGATTGGGAAGAgAATAGCAGGCATGCTGGGGATAGTTATTAATAGTAATC
AATTACGGGGTCATTAGTTCATAGCCCATATATGGAGTTCCGCGTTACATAACTTACGGTAAATGGCCCGCCTGGCTGACCGCCCAACGACCCCCGCCCATTGACGTCAATAATGACGTATGTTCCCATAGTAACGCCAATAGGGACTTTCCATTGACGTCAATGGGTGGAGTATTTACGGTAAACTGCCCACTTGGCAGTACATCAAGTGTATCATATGCCAAGTACGCCCCCTATTGACGT
CAATGACGGTAAATGGCCCGCCTGGCATTATGCCCAGTACATGACCTTATGGGACTTTCCTACTTGGCAGTACATCTACGTATTAGTCATCGCTATTACCATGGTGATGCGGTTTTGGCAGTACATCAATGGGCGTGGATAGCGGTTTGACTCACGGGGATTTCCAAGTCTCCACCCCATTGACGTCAATGGGAGTTTGTTTTGGCACCAAAATCAACGGGACTTTCCAAAATGTCGTAACA
ACTCCGCCCCATTGACGCAAATGGGCGGTAGGCGTGTACGGTGGGAGGTCTATATAAGCAGAGCTGGTTTAGTGAACCGTCAGATCCgccaccATGGTGAGCAAGGGCGAGGAGCTGTTCACCGGGGTGGTGCCCATCCTGGTCGAGCTGGACGGCGACGTAAACGGCCACAAGTTCAGCGTGTCCGGCGAGGGCGAGGGCGATGCCACCTACGGCAAGCTGACCCTGAAGTT
CATCTGCACCACCGGCAAGCTGCCCGTGCCCTGGCCCACCCTCGTGACCACCCTGACCTACGGCGTGCAGTGCTTCAGCCGCTACCCCGACCACATGAAGCAGCACGACTTCTTCAAGTCCGCCATGCCCGAAGGCTACGTCCAGGAGCGCACCATCTTCTTCAAGGACGACGGCAACTACAAGACCCGCGCCGAGGTGAAGTTCGAGGGCGACACCCTGGTGAACCGCAT
CGAGCTGAAGGGCATCGACTTCAAGGAGGACGGCAACATttcaGGGCACAAGCTGGAGTACAACTACAACAGCCACAACGTCTATATCATGGCCGACAAGCAGAAGAACGGCATCAAGGTGAACTTCAAGATCCGCCACAACATCGAGGACGGCAGCGTGCAGCTCGCCGACCACTACCAGCAGAACACCCCCATCGGCGACGGCCCCGTGCTGCTGCCCGACAACCACTACCT
GAGCACCCAGTCCGCCCTGAGCAAAGACCCCAACGAGAAGCGCGATCACATGGTCCTGCTGGAGTTCGTGACCGCCGCCGGGATCACTCTCGGCATGGACGAGCTGTACAAGGCTACTAACTTCAGCCTGCTGAAGCAGGCTGGAGACGTGGAGGAGAACCCTGGACCTGCTAGcgccaccatggtgagcaagggcgaggaggataacatggccatcatcaaggagttcatgcgcttcaaggtgcacatgg
agggctccgtgaacggccacgagttcgagatcgagggcgagggcgagggccgcccctacgagggcacccagaccgccaagctgaaggtgaccaagggtggcccccctgcccttcgcctgggacatcctgtcccctcagttcatgtacggctccaaggcctacgtgaagcaccccgccgacatccccgactacttgaagctgtccttccccgagggcttcaagtgggagcgcgtgatgaacttcgaTgacggcggcgtggtgaccgtgacccaggactcctccctgcaggacggcgagttcatctacaaggtg
aagctgcgcggcaccaacttcccctccgacggccccgtaatgcagaagaaAaccatgggctgggaggcctcctccgagcggatgtaccccgaggacggcgccctgaagggcgagatcaagcagaggctgaagctgaaggacggcggccactacgacgctgaggtcaagaccacctacaaggccaagaagcccgtgcagctgcccggcgcctacaacgtcaacatcaagttggacatcacctcccacaacgaggactacaccatcgtggaacagtacgaacgcgccgagggccgccactccaccgg
cggcatggacgagctgtacaagtaactcttggcccGGATCCctgtgccttctagttgccagccatctgttgtttgcccctcccccgtgccttccttgaccctggaaggtgccactcccactgtcctttcctaataaaatgaggaaattgcatcgcattgtctgagtaggtgtcattctattctggggggtggggtggggcaggacagcaagggggaggattgggaaAacaatagcaggcatgctggggatgcggtgggctctatgg

pEF1a-gRNA-Ec73 ncRNA-HDV-polyA-pCMV-mCherry-polyA

ggctccggtgcccgtcagtgggcagagcgcacatcgcccacagtccccgagaagttggggggaggggtcggcaattgaaccggtgcctagagaaggtggcgcggggtaaactgggaaagtgatgtcgtgtactggctccgcctttttcccgagggtgggggagaaccgtatataagtgcagtagtcgccgtgaacgttctttttcgcaacgggtttgccgccagaacacaggtaagtgccgtgtgtggttcccgcgggcctggcctctttacgggttatggcccttgcgtgccttgaattacttccacctggctgcagta
cgtgattcttgatcccgagcttcgggttggaagtgggtgggagagttcgaggccttgcgcttaaggagccccttcgcctcgtgcttgagttgaggcctggcctgggcgctggggccgccgcgtgcgaatctggtggcaccttcgcgcctgtctcgctgctttcgataagtctctagccatttaaaatttttgatgacctgctgcgacgctttttttctggcaagatagtcttgtaaatgcgggccaagatctgcacactggtatttcggtttttggggccgcgggcggcgacggggcccgtgcgtcccagcgcacatgttcggcga
ggcggggcctgcgagcgcggccaccgagaatcggacgggggtagtctcaagctggccggcctgctctggtgcctggcctcgcgccgccgtgtatcgccccgccctgggcggcaaggctggcccggtcggcaccagttgcgtgagcggaaagatggccgcttcccggccctgctgcagggagctcaaaatggaggacgcggcgctcgggagagcgggcgggtgagtcacccacacaaaggaaaagggcctttccgtcctcagccgtcgcttcatgtgactccacggagtaccgggcgccgtccaggca
cctcgattagttctcgagcttttggagtacgtcgtctttaggttggggggaggggttttatgcgatggagtttccccacactgagtgggtggagactgaagttaggccagcttggcacttgatgtaattctccttggaatttgccctttttgagtttggatcttggttcattctcaagcctcagacagtggttcaaagtttttttcttccatttcaggtgtcgtgaacgcgtggGTCTTCgaGAAGACCtgttttagagctagaaatagcaagttaaaataaggctagtccgttatcaacttgaaaaagtggcaccgagtcggtgcCGC
CAGCAGTGGCAATAGCGTTTCCGGCCTTTTGTGCCGGGAGGGTCGGCGAGTCGCTGACTTAACGCCAGTAGTATGTCCATgaattcATACCCAAAGgaattcATGGTTTAATGGTATTGCCGCTGTTGGCGggccggcatggtcccagcctcctcgctggcgccggctgggcaacaTGcttcggCAtggcgaatgggacacgcgtCAGCCTCGACTGTGCCTTCTAGTTGCCAGCCATCTGTTGTTTGCCCCTCCCCC
GTGCCTTCCTTGACCCTGGAAGGTGCCACTCCCACTGTCCTTTCCTAATAAAATGAGGAAATTGCATCGCATTGTCTGAGTAGGTGTCATTCTATTCTGGGGGGTGGGGTGGGGCAGGACAGCAAGGGGGAGGATTGGGAAGAgAATAGCAGGCATGCTGGGGATAGTTATTAATAGTAATCAATTACGGGGTCATTAGTTCATAGCCCATATATGGAGTTCCGCGTTACATAACTTACGGT
AAATGGCCCGCCTGGCTGACCGCCCAACGACCCCCGCCCATTGACGTCAATAATGACGTATGTTCCCATAGTAACGCCAATAGGGACTTTCCATTGACGTCAATGGGTGGAGTATTTACGGTAAACTGCCCACTTGGCAGTACATCAAGTGTATCATATGCCAAGTACGCCCCCTATTGACGTCAATGACGGTAAATGGCCCGCCTGGCATTATGCCCAGTACATGACCTTATGGGACTTT
CCTACTTGGCAGTACATCTACGTATTAGTCATCGCTATTACCATGGTGATGCGGTTTTGGCAGTACATCAATGGGCGTGGATAGCGGTTTGACTCACGGGGATTTCCAAGTCTCCACCCCATTGACGTCAATGGGAGTTTGTTTTGGCACCAAAATCAACGGGACTTTCCAAAATGTCGTAACAACTCCGCCCCATTGACGCAAATGGGCGGTAGGCGTGTACGGTGGGAGGTCTATATAA
GCAGAGCTGGTTTAGTGAACCGTCAGATCCgccaccATGGTGAGCAAGGGCGAGGAGCTGTTCACCGGGGTGGTGCCCATCCTGGTCGAGCTGGACGGCGACGTAAACGGCCACAAGTTCAGCGTGTCCGGCGAGGGCGAGGGCGATGCCACCTACGGCAAGCTGACCCTGAAGTTCATCTGCACCACCGGCAAGCTGCCCGTGCCCTGGCCCACCCTCGTGACCACCCTGA
CCTACGGCGTGCAGTGCTTCAGCCGCTACCCCGACCACATGAAGCAGCACGACTTCTTCAAGTCCGCCATGCCCGAAGGCTACGTCCAGGAGCGCACCATCTTCTTCAAGGACGACGGCAACTACAAGACCCGCGCCGAGGTGAAGTTCGAGGGCGACACCCTGGTGAACCGCATCGAGCTGAAGGGCATCGACTTCAAGGAGGACGGCAACATCCTGGGGCACAAGCTGG
AGTACAACTACAACAGCCACAACGTCTATATCATGGCCGACAAGCAGAAGAACGGCATCAAGGTGAACTTCAAGATCCGCCACAACATCGAGGACGGCAGCGTGCAGCTCGCCGACCACTACCAGCAGAACACCCCCATCGGCGACGGCCCCGTGCTGCTGCCCGACAACCACTAttcaAGtACCCAGTCgGCCCTGAGCAAAGACCCCAACGAGAAGCGCGATCACATGGTCCT
GCTGGAGTTCGTGACCGCCGCCGGGATCACTCTCGGCATGGACGAGCTGTACAAGGCTACTAACTTCAGCCTGCTGAAGCAGGCTGGAGACGTGGAGGAGAACCCTGGACCTGCTAGcgccaccatggtgagcaagggcgaggaggataacatggccatcatcaaggagttcatgcgcttcaaggtgcacatggagggctccgtgaacggccacgagttcgagatcgagggcgagggcgagggccgcccctacgagggcacccagacc
gccaagctgaaggtgaccaagggtggcccccctgcccttcgcctgggacatcctgtcccctcagttcatgtacggctccaaggcctacgtgaagcaccccgccgacatccccgactacttgaagctgtccttccccgagggcttcaagtgggagcgcgtgatgaacttcgaTgacggcggcgtggtgaccgtgacccaggactcctccctgcaggacggcgagttcatctacaaggtgaagctgcgcggcaccaacttcccctccgacggccccgtaatgcagaagaaAaccatgggctgggaggcctcctccg
agcggatgtaccccgaggacggcgccctgaagggcgagatcaagcagaggctgaagctgaaggacggcggccactacgacgctgaggtcaagaccacctacaaggccaagaagcccgtgcagctgcccggcgcctacaacgtcaacatcaagttggacatcacctcccacaacgaggactacaccatcgtggaacagtacgaacgcgccgagggccgccactccaccggcggcatggacgagctgtacaagtaactcttggcccGGATCCctgtgccttctagttgccagccatctgttgtttgcccc
tcccccgtgccttccttgaccctggaaggtgccactcccactgtcctttcctaataaaatgaggaaattgcatcgcattgtctgagtaggtgtcattctattctggggggtggggtggggcaggacagcaagggggaggattgggaaAacaatagcaggcatgctggggatgcggtgggctctatgg

Supplemental Table1. Primers and oligos used in this study

Primers/Oligos for cloning		
**Primer Name**	**Primers Sequences**	
Cas9-Ec86RT F	gcagcagcggggggtcaAGCAAAAGCGCCGAGTACCTGAATAC	Retron RTs fused to the carboxy terminus of Cas9
Cas9-Ec86RT R	GGTTCTTTTTGAGCCGCCAGAGGTCTTAGCTTTGTTCAGAGGATTC	
Cas9-Ec48RT F	ggcagcagcggggggtcaAGCGGCAGACCTTACGTGACCCTG	
Cas9-Ec48RT R	GGTTCTTTTTGAGCCGCCAGACAGGGGCTTGAGGCTGGCCAG	
Cas9-Ec73RT F	ggcagcagcggggggtcaAGCCGGATCTACAGCCTGATTGATAG	
Cas9-Ec73RT R	GGTTCTTTTTGAGCCGCCAGATTCCACTTTGTTGTGCAGGTTC	
Cas9-Ec107RT F	ggcagcagcggggggtcaAGCGACGCCACAAGAACCACCCTG	
Cas9-Ec107RT R	GGTTCTTTTTGAGCCGCCAGACCAGGCCACCAGCATCCGTTTC	
Ec86RT-Cas9 F	ATCCACGGAGTCCCAGCAGCCcccgggAAAAGCGCCGAGTACCTGAATAC	Retron RTs fused to the amino terminus of Cas9
Ec86RT-Cas9 R	cctccgctagatcctccggacccgggGGTCTTAGCTTTGTTCAGAGGATTC	
Ec48RT-Cas9 F	ATCCACGGAGTCCCAGCAGCCcccgggGGCAGACCTTACGTGACCCTGAAC	
Ec48RT-Cas9 R	gagcctccgctagatcctccggacccgggCAGGGGCTTGAGGCTGGCCAG	
Ec73RT-Cas9 F	TATCCACGGAGTCCCAGCAGCCcccgggCGGATCTACAGCCTGATTGATAG	
Ec73RT-Cas9 R	agcctccgctagatcctccggacccgggTTCCACTTTGTTGTGCAGGTTC	
Ec107RT-Cas9 F	TATCCACGGAGTCCCAGCAGCCcccgggGACGCCACAAGAACCACCCTGCTG	
Ec107RT-Cas9 R	agcctccgctagatcctccggacccgggCCAGGCCACCAGCATCCGTTTC	
oligo template for *EMX1* donor	AAACGGCAGAAGCTGGAGGAGGAAGGGCCTGAGTCCGAGCAGAAGAAGCTTAAGGGCTCCCATCACATCAACCGGTGGCGCATTGCCACGAAGCAG	PCR template
oligo template for *HEK3* donor with BcuI insertion	cttctccagccctggcctgggtcaatccttggggcccagactgagcactaGtgatggcagaggaaaggaagccctgcttcctccagagggcgtcgcag	
oligo template for *HEK3* donor with G to T transversion	cttctccagccctggcctgggtcaatccttggggcccagactgagcacttgatggcagaggaaaggaagccctgcttcctccagagggcgtcgcag	
Ec86-*EMX1* donor F	atctgagttactgtctgtttccctACTAGTACAAACGGCAGAAGCTGGAGGA	Expressing 5' rgRNA and 3' rgRNA containing *EMX1* donor sequences
Ec86-*EMX1* donor R	CGCACCCTTACGTCAGAAgAAACGGGTTcCCTcctaggTGACATCGATGTCCTCCCCATTGGCCTGCTTCGTGGCAATGC	
Ec48-*EMX1* donor F	CAGCGAGGCCCCACCGTCGTCAACTAGTACAAACGGCAGAAGCTGGAGGA	
Ec48-*EMX1* donor R	GGCTACGGCACCATCGTAGTcctaggTGACATCGATGTCCTCCCCATTGGCCTGCTTCGTGGCAATGC	
Ec73-*EMX1* donor F	TATTTTAGCTATGCCCGTCGTTACTAGTACAAACGGCAGAAGCTGGAGGA	
Ec73-*EMX1* donor R	AACCTACTTGAGCACGTCGATcctaggTGACATCGATGTCCTCCCCATTGGCCTGCTTCGTGGCAATGC	
Ec107-*EMX1* donor F	TTAACGCCAGTAGTATGTCCATACTAGTACAAACGGCAGAAGCTGGAGGA	
Ec107-*EMX1* donor R	AGCGGCAATACCATTAAACCATcctaggTGACATCGATGTCCTCCCCATTGGCCTGCTTCGTGGCAATGC	
Ec86-*HEK3* donor F	aatctgagttactgtctgtttccctACTAGTgcttctccagccctggcctgggt	Expressing 5' rgRNA and 3' rgRNA containing *HEK3* donor sequences
Ec86-*HEK3* donor R	GCGCACCCTTACGTCAGAAgAAACGGGTTcCCTcctagggcccctgtctaggaaaagctgtcctgcgacgccctctggag	
Ec48-*HEK3* donor F	AGCGAGGCCCCACCGTCGTCAACTAGTgcttctccagccctggcctg	
Ec48-*HEK3* donor R	GTCGGCTACGGCACCATCGTAGTcctagggcccctgtctaggaaaagctgtcctgcgacgccctctggag	
Ec73-*HEK3* donor F	TAGTATTTTAGCTATGCCCGTCGTTACTAGTgcttctccagccctggcctg	
Ec73-*HEK3* donor R	AAACCTACTTGAGCACGTCGATcctagggcccctgtctaggaaaagctgtcctgcgacgccctctggag	
Ec107-*HEK3* donor F	ACTTAACGCCAGTAGTATGTCCATACTAGTgcttctccagccctggcctg	
Ec107-*HEK3* donor R	CAGCGGCAATACCATTAAACCATcctagggcccctgtctaggaaaagctgtcctgcgacgccctctggag	

Supplemental Table1. Primers and oligos used in this study

**Primers for genomic DNA amplification**		
EMX1 F1	AAAACCACCCTTCTCTCTGGC	For RFLP
EMX1 R1	GGAGATTGGAGACACGGAGAG	
EMX1 F2	CCATCCCCTTCTGTGAATGT	
EMX1 R2	GGAGATTGGAGACACGGAGA	
HEK3 F1	tggcaaatgaggctggagagg	
HEK3 R1	cagagctgtcctctggttctg	
HEK3 F2	ggcatgagaaaccttggaga	
HEK3 R2	cagagctgtcctctggttct	
EMX1 DSeq F1	CCATCCCCTTCTGTGAATGT	For deep sequencing
EMX1 DSeq R1	TaGCCTGTTTCTCCTGCTGT	
EMX1 DSeq F2	gaattcNNNNNNNNggatccCAGGTGAAGGTGTGGTTCCAG	
EMX1 DSeq R2	gaattcNNNNNNNNggatccCCCTCGTGGGTTTGTGGTTG	
HEK3 DSeq F1	tggcaaatgaggctggagagg	
HEK3 DSeq R1	cttagctggcatggcaatgtctc	
HEK3 DSeq F2	gaattcNNNNNNNNggatccCTGCTGCAAGTAAGCATGCA	
HEK3 DSeq R2	gaattcNNNNNNNNggatccGCCCAGCCAAACTTGTCAAC	
EMX1 OT1 TIDE F1	GACAGCTTCTTATCCCTGTCTTCCAG	For off-target analysis by TIDER assay
EMX1 OT1 TIDE R1	TTCCATTTCTCTGCATCACCATTCTG	
EMX1 OT1 TIDE F2	ATTCCTGAAGACCTGTAATCTGACTC	
EMX1 OT1 TIDE R2	TTCTCTGCATCACCATTCTGGTC	
EMX1 OT2 TIDE F1	GATGAATCCTCCTCCTGTTCCTCAG	
EMX1 OT2 TIDE R1	GTAAGAGATTACATACTCTTGCATGTTTG	
EMX1 OT2 TIDE F2	CATCCTCCTCATCTGAGGACTCCA	
EMX1 OT2 TIDE R2	TTGCATGTTTGATAACACATAAGGTAAAGG	
EMX1 OT3 TIDE F1	CAAAGGAGACCTGGAGACGACTG	
EMX1 OT3 TIDE R1	GACCCCAGCTCACCTCACTC	
EMX1 OT3 TIDE F2	AGGCACTTGCGGTTTGCACTG	
EMX1 OT3 TIDE R2	CCCTTCCGGTTGCCCTTCAC	
EMX1 OT4 TIDE F1	aagaagccataccgagcggttg	
EMX1 OT4 TIDE R1	tgtgtacagagtttctcatgtgaccttctg	
EMX1 OT4 TIDE F2	gaagccataccgagcggttgG	
EMX1 OT4 TIDE R2	tacagagtttctcatgtgaccttctgatc	
**Primers for testing expression of msDNA**		
HEK3 FP	gcttctccagccctggcctg	
HEK3 RP	gcccctgtctaggaaaagctgtc	
